# Case Report: Large ileal intramural hematoma presenting as an intestinal obstruction in a patient on Warfarin with incidental breast cancer

**DOI:** 10.12688/f1000research.14848.2

**Published:** 2019-12-30

**Authors:** Maryam Alizadeh Forutan, Fereshteh Araghian Mojarad, Nasrin Rahmani

**Affiliations:** 1 Gastrointestinal Cancer Research Center, Mazandaran University of Medical Science, Sari, Iran; 2 Golestan University of Medical Sciences, Gorgan, Iran; 3 Department of General Surgery, Faculty of Medicine, Mazandaran University of Medical Sciences, Sari, Iran

**Keywords:** Gastrointestinal Tract, hematoma, anticoagulant therapy, warfarin.

## Abstract

Intramural hematoma of the gastrointestinal (GI) tract, which can present as abdominal pain or obstruction, can be a rare complication of oral anticoagulants, in particular Warfarin. In this case report, we describe an 81-year-old female patient presenting with abdominal pain, nausea, and vomiting with a previous history of rectorrhagia. The patient was receiving Warfarin therapy due to cardiac valve replacement for the past 8 years. Laboratory workup revealed elevated INR and anemia. Diagnosis of ileal intramural hematoma was based on ultrasound and CT scan findings. The patient was treated by conservative approaches including administration of fresh frozen plasma, cessation of oral intake, and fluid resuscitation. In CT images, a mass on the left breast and lymphadenopathy on the left axilla were also noticed. Given that most GI intramural hematomas caused by over-anticoagulation are treated non-surgically, considering a patient's drug history, especially in older patients with abdominal pain and obstruction symptoms, is of particular importance.

## Introduction

Abdominal pain is a common complaint in patients referred to hospital emergency departments and varies from mild and self-limiting to severe and life-threatening conditions ^1, 2^. Depending on the location of the pain, symptoms, physical examination and medical history, a differential diagnosis is provided ^1^. Since mortality and the rate of surgery secondary to abdominal pain is higher in elderly patients, prompt diagnosis of the causative agent is of particular importance ^3^. Non-specific causes, gastroenteritis, irritable bowel syndrome, urologic disorders, and gastroenteritis are among the common etiologies for abdominal pain ^4^. An uncommon reason for abdominal pain and bowel obstruction is intestinal intramural hematoma following anticoagulation therapy ^5, 6^. Warfarin, a vitamin K antagonist, is widely used to prevent thrombosis formation due to mechanical heart valves, atrial fibrillation, pulmonary embolism and deep venous thrombosis ^7^. The occurrence of intestinal intramural hematoma secondary to anticoagulant therapy is an uncommon condition, which affects 1/2500 patients receiving Warfarin ^8^. The small intestine is the common site affected by spontaneous intramural hematoma, and intramural hematoma of the colon and rectum are rare ^9^. This complication is mostly treated by non-surgical approaches.

This article reports a case of a relatively large ileal intramural hematoma developed following Warfarin use and accidental detection of breast malignancy during a CT scan.

## Case presentation

An 81-year-old woman was admitted to the Mazandaran Heart Center, Sari, Iran in February, 2018 with a 4-day history of nausea, vomiting, and abdominal pain. She had been taking Warfarin (5 mg orally once a day) and aspirin (80 mg/day) for 8 years after aortic valve replacement without close monitoring and had a history of hematochezia caused by Warfarin toxicity one year ago. Colicky pain, an increase in bowel sounds and periumbilical tenderness without distension was determined by physical examination. The primary laboratory findings revealed anemia (Hb: 9.8 g/dl; normal value: 11.5–13.5 g/dl) and elevated INR (6; therapeutic range: 2–3.5), liver function and biochemistry tests were within normal values. Normal left ventricular systolic function, LVEF of 55–60%, septal hypertrophy, normal functioning prosthetic valve, and dilatation of the ascending aorta were reported by echocardiography.

During an abdominal and pelvic ultrasound, long mucosal thickening possibly through the ascending and sigmoid colon was observed, suggestive of intramural hematoma. The patient was placed on *nil per os*, received supportive care and two units of fresh frozen plasma. Due to normal cardiac evaluation and partial relief of symptoms on the second day of admission, the patient was referred to surgical consultation with the possible diagnosis of descending colon and sigmoid intramural hematoma. For re-administration of anticoagulants, heparin infusion (1000 units/hour) began with precise monitoring of prothrombin time.

**Figure 1.**
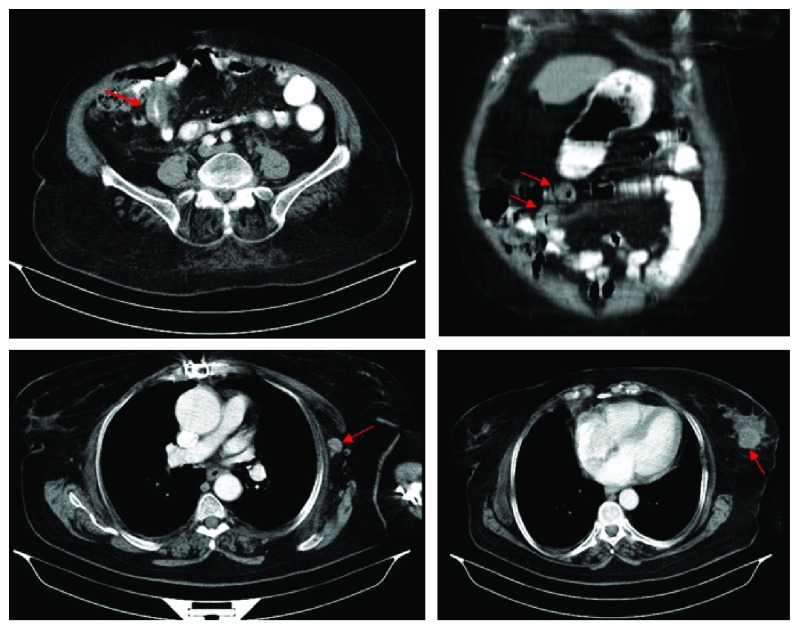
Contrast-enhanced abdominal and pelvic CT. (A) Circumferential thickening of an ileal loop with adjacent fat stranding in Sagittal (1) and Coronal (2) view. (B) LAP on left axilla with malignant feature measuring 18×16mm (3) and 32×26 mm mass in left breast (4).

On the fourth day, CT scan was performed with intravenous and oral contrast ( Figure 1) and intramural hematoma of ileum was diagnosed. During the CT, a radiologist noticed a mass on the left breast and a lymph node with a malignant feature on the left axilla. With the improvement of abdominal pain and vomiting (on the fourth day), oral feeding was resumed. After mammography and breast biopsy, pathological examination revealed invasive ductal cell carcinoma with lymphatic and perineural invasion. According to the immunohistochemistry staining, tumor cells were strongly ER positive, PR negative, 15% Ki67 positive and equivocal for HER2. The patient was referred to the oncology-hematology department to receive appropriate treatment.

The patient was discharged 10 days after admission and Warfarin therapy (5 mg/d) was resumed. The patient was visited 2 weeks later with good general health assessment. Letrozole (2.5 mg/day) was started because the patient refused chemotherapy and radiotherapy.

## Discussion

The most significant reason for the intestinal intramural hematoma is trauma, while non-traumatic or spontaneous causes include anticoagulation therapy, malignancies, and blood disorders, which are considered to be rare ^10, 11^. The intestinal sites involved in the intramural hematoma in order of frequency include jejunum, duodenum, and ileum ^12^. The intramural hematoma can affect the esophagus, gastric and colon, but these cases are less prevalent. This complication is mostly associated with Warfarin and is more common in male subjects ^10, 13, 14^; patient's chief complaints are abdominal pain, nausea, vomiting, and absence of bowel movements or flatulence ^6, 15^. Melena and jaundice are rare presentation too ^16^. Hyperechoic bowel wall thickening or free fluid may be noted in ultrasound findings, nevertheless, as a non-unspecific test, normal ultrasound results cannot rule out the possible diagnosis of gastrointestinal intramural hematoma. The diagnostic key test is a CT scan, but there is no agreement in the literature concerning the use of contrast materials, as it may obscure intramural hyper-density and hemorrhage, and also increase the risk of exposure ^17, 18^. Bowel wall thickening, luminal narrowing, obstruction caused by edema, picket fence sign can be demonstrated in CT-scan ^19, 20^. Products related to hemoglobin breakdown in hematoma site will lead to a high density signal as “ring sign” in MRI which is unique to confirm diagnosis ^21^. In the review of fourteen patients by Yoldaş *et al*, nine patients were treated non-surgically, with eight of them were on Warfarin therapy for heart disorders. Increased INR was also observed in anticoagulant receiving patients ^10^. In addition to impaired coagulation tests, increased WBC count and anemia may also present ^15, 19^. For early diagnosis of this complication, taking a detailed medical history of anticoagulation use, especially in older patients with abdominal pain, is required. Treatment usually is conservative and includes cessation of oral anticoagulation, serum therapy, and correction of coagulation indices by vitamin K and blood products ^8, 19, 22^. As this condition is rare, there is no treatment as the best option in the literature but unlike the past, there is a tendency for conservative management of cases today. The patient's condition is the most important consideration in choosing the treatment. Referral to surgery is usually preferred when the patient has peritonitis, obstruction, ischemia or necrosis ^16^. Accurate recognition of this complication leads to prevention of unnecessary surgical procedures and the risk of bleeding progression. There are also evidence that Warfarin may act as an anticancer agent; Warfarin was shown to lower the incidence of organ specific cancers including lung, prostate, and breast ^23, 24^. Therefore, the development of invasive breast cancer in the present case, despite the long-term use of Warfarin, is interesting.

Finally, the question arises of which anticoagulant can be used as an alternative to Warfarin in patients with frequent life threatening hemorrhagic events, which requires no regular monitoring but has an acceptable efficacy.

## Consent

Written informed consent was obtained from patient during admission and follow up for the publication of the patient’s clinical information and accompanying images.

## Data availability

All data underlying the results are available as part of the article and no additional source data are required.
