# Supplementary material for: Case Report: Large ileal intramural hematoma presenting as an intestinal obstruction in a patient on Warfarin with incidental breast cancer
Source: F1000Res. 2019 Dec 30;7:724. Originally published 2018 Jun 11. [Version 2] doi: 10.12688/f1000research.14848.2 (PMC7059841; doi:10.12688/f1000research.14848.2)
Supplement: Peer review history

# Peer review: Reviewer response for version 2

**DOI:** 10.5256/f1000research.23800.r58052

**Published:** 2020-03-02

**Authors:** Everton Cazzo

**Affiliations:** 1 Department of Surgery, Faculdade de Ciências Médicas da Universidade Estadual de Campinas (FCM/UNICAMP), Campinas, Brazil

All the comments and suggestions were appropriately addressed.

I confirm that I have read this submission and believe that I have an appropriate level of expertise to confirm that it is of an acceptable scientific standard.

# Peer review: Reviewer response for version 1

**DOI:** 10.5256/f1000research.16162.r55318

**Published:** 2019-10-21

**Authors:** Everton Cazzo

**Affiliations:** 1 Department of Surgery, Faculdade de Ciências Médicas da Universidade Estadual de Campinas (FCM/UNICAMP), Campinas, Brazil

This is an interesting and well-written case report of a disease which poses a significant difficulty in relation to its diagnosis and management.

 The main comments which I think may improve and enrich this study are the following:

1. The discussion should include a more profound comment on the therapeutic options available for the cases when the bleeding does not stop after interrupting Warfarin, mainly radiological and surgical interventions.
2. The discussion should also include some comments on hematomas which arise on other segments of the gastrointestinal tract, such as stomach and duodenum, since they are close to the topic reported.

I confirm that I have read this submission and believe that I have an appropriate level of expertise to confirm that it is of an acceptable scientific standard, however I have significant reservations, as outlined above.

# Response

**Published:** 2019-11-05

**Authors:** Maryam Alizadeh Forutan

**Affiliations:** Medical Sciences University of Mazandaran, Iran

Hello and thanks for your attention.

 I will definitely consider and correct the things you have mentioned.

# Response

**Published:** 2019-12-10

**Authors:** Maryam Alizadeh Forutan

**Affiliations:** Medical Sciences University of Mazandaran, Iran

Dear reviewer,

Many thanks for your attention and points.

We have revised the manuscript according to your suggestions and highlighted them.

# Peer review: Reviewer response for version 1

**DOI:** 10.5256/f1000research.16162.r35009

**Published:** 2019-09-12

**Authors:** Kevin W. Olden

**Affiliations:** 1 Division of Gastroenterology, Regional One Medical Center, University of Tennessee, School of Medicine, Memphis, TN, USA

- The case is well presented, and the clinical details are well written and meticulously enumerated. The case is relevant for practice and will benefit clinicians. 
- The only comment I would have is to change the word "ileum" on p.2 (2nd paragraph) to "ileal" and the word "rectorrhagia" to "hematochezia" in order to conform to the more commonly used medical terminology.
- This paper is an excellent case report that presents useful information for the clinician. I would recommend acceptance if the two semantic changes were made.

I confirm that I have read this submission and believe that I have an appropriate level of expertise to confirm that it is of an acceptable scientific standard.

# Response

**Published:** 2019-09-14

**Authors:** Maryam Alizadeh Forutan

**Affiliations:** Medical Sciences University of Mazandaran, Iran

Greetings and regards.

Thanks for bothering to read this case report and for taking the time to review the article.

I will consider the issues raised.
